# The molecular mechanisms underpinning maternal mRNA dormancy

**DOI:** 10.1042/BST20231122

**Published:** 2024-03-13

**Authors:** Laura Lorenzo-Orts, Andrea Pauli

**Affiliations:** Research Institute of Molecular Pathology (IMP), Vienna BioCenter (VBC), 1030 Vienna, Austria

**Keywords:** embryogenesis, mRNA, mRNA stability, oogenesis, translation

## Abstract

A large number of mRNAs of maternal origin are produced during oogenesis and deposited in the oocyte. Since transcription stops at the onset of meiosis during oogenesis and does not resume until later in embryogenesis, maternal mRNAs are the only templates for protein synthesis during this period. To ensure that a protein is made in the right place at the right time, the translation of maternal mRNAs must be activated at a specific stage of development. Here we summarize our current understanding of the sophisticated mechanisms that contribute to the temporal repression of maternal mRNAs, termed maternal mRNA dormancy. We discuss mechanisms at the level of the RNA itself, such as the regulation of polyadenine tail length and RNA modifications, as well as at the level of RNA-binding proteins, which often block the assembly of translation initiation complexes at the 5′ end of an mRNA or recruit mRNAs to specific subcellular compartments. We also review microRNAs and other mechanisms that contribute to repressing translation, such as ribosome dormancy. Importantly, the mechanisms responsible for mRNA dormancy during the oocyte-to-embryo transition are also relevant to cellular quiescence in other biological contexts.

## Introduction

Immediately after fertilization, embryos rely on components of maternal origin for development. In metazoans, transcription stops at the onset of meiosis during oogenesis and resumes only with zygotic genome activation (ZGA) in the embryo. The time between oocyte meiotic arrest and ZGA reflects the period during which maternal mRNAs are in sole control of gene expression, and it varies widely among species. The ovaries of adult fish, frogs, and flies contain mitotic germ cells (oogonia) that are likely to maintain oogenesis throughout adulthood [1]. In contrast, the ovaries of adult mammals are depleted of oogonia and contain a fixed number of meiotically arrested oocytes that were produced during embryogenesis (weeks or decades ago) [1]. The timing of ZGA also varies between species: In human and mouse embryos, ZGA occurs after 1 or 2 cell cycles, corresponding to ∼24 and 44 h after fertilization (hpf), respectively [2]. In contrast, flies, fish, and frogs undergo several cell cycles before ZGA, namely 13 in *Drosophila melanogaster* (∼2 hpf), 10 in *Danio rerio* (∼3 hpf), and 6 in *Xenopus tropicalis* (∼4 hpf). [2]. Despite the differences in oogenesis and ZGA for each species, it is clear that maternal mRNAs must be stored for periods of time well beyond the estimated 3–4 h average half-life of mRNAs in somatic cells [3]. For example, 90% of the mRNAs produced during zebrafish oogenesis are still present in gastrulating embryos after 5 hpf [4]. In mouse oocytes, studies in the early 1980s using radiolabeled RNA suggested mRNA half-lives of several days or weeks [5,6], and mouse embryonic stem cell mRNAs have an estimated median half-life of 7.1 h [7].

Since all maternal mRNAs are produced during oogenesis before transcription is turned off, translation must be tightly regulated to ensure that proteins are made at the right place and time. Several studies in different organisms indicate that translation is repressed in the mature oocyte and gradually increases during embryogenesis [8–10]. We refer to the temporal translational repression of maternal mRNAs as maternal mRNA dormancy. To achieve this, maternal mRNAs undergo reversible changes in RNA modifications and polyA tail length, and transiently associate with RNA-binding proteins or microRNAs (miRNAs). Furthermore, maternal mRNAs can localize to specialized RNA granules that are important for mRNA storage and localization. Here, we review the basic principles regulating maternal mRNA dormancy and summarize open questions. For translational dynamics during the oocyte-to-embryo transition, we refer to other excellent reviews [2,11,12]. Importantly, insights gained by studying the post-transcriptional mechanisms governing the oocyte-to-embryo transition have led to key discoveries in RNA biology, such as the importance of the polyA tail for translation.

## Polyadenine tail length regulation

Maternal mRNAs undergo changes in their polyA tail lengths during development, as demonstrated in the 1970s when mRNAs in the cytoplasm of unfertilized sea urchin eggs were found to have short or no polyA tails, whereas mRNAs in fertilized eggs and embryos had long tails [13]. This is striking because short polyA tails have been associated with mRNA degradation in somatic cells [14], whereas maternal mRNAs are particularly stable [4–7]. A decade later, differences in the polyA tail length of maternal mRNAs were associated with changes in their translatability [15]. Measurement of translational efficiency of maternal mRNAs became possible with the development of ribosome profiling [16]. In contrast to somatic mRNAs, maternal mRNAs showed a strong correlation between polyA tail length and translation during early stages of embryogenesis [17].

**Figure 1.**
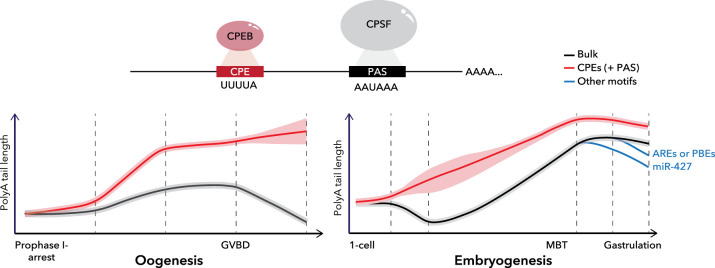
Changes in polyA tail length during oogenesis and embryogenesis. Maternal mRNAs are deadenylated by default during oogenesis in several species, including flies [21], mice [22], and frogs [18,19], unless they contain cytoplasmic polyadenylation elements (CPEs) and/or polyadenylation signals (PASs) recognized by CPEB and CPSF (top). According to [20], mRNA deadenylation occurs after germinal vesicle breakdown (GVBD) in frog oocytes (left). In the embryo (right), recent work suggests that mRNAs are repolyadenylated after the first few cleavages in frog and fish [20]. Deadenylation in the embryo is influenced by motifs, including AU-rich elements (AREs) and Pumilio binding elements (PBE) [20].

How is the polyA tail of maternal mRNAs regulated to reach specific lengths during development? To prepare the oocyte for future quiescence, maternal transcripts are deadenylated by default during oogenesis and early embryogenesis [18–20] (Figure 1). Genetic and functional experiments in zebrafish [23] and Xenopus [24] suggest that the polyA-specific ribonuclease PARN plays a critical role in this process. However, certain mRNAs containing specific motifs in the 3′ untranslated region (UTR), namely a polyadenylation signal (PAS) and a cytoplasmic polyadenylation element (CPE), can escape PARN-mediated deadenylation in the oocyte. The PAS, consisting of AAUAAA or AUUAAA, is required for both nuclear and cytoplasmic polyadenylation and is recognized by the Cleavage and Polyadenylation Specificity Factor (CPSF) [25]. In contrast, CPE consists of a U-rich sequence that is only involved in cytoplasmic polyadenylation and is recognized by CPEB [26]. CPEB-mediated polyadenylation can be influenced by specific mRNA features, including the number of CPEs and PAS, as well as the distance between PAS and CPE or between PAS and the polyA site [20,27]. While CPE is the main element contributing to cytoplasmic polyadenylation in frog [20] and mouse [22] oocytes, other motifs have also been implicated in polyA tail length regulation (reviewed in [28]). In particular, mRNAs that require early translation in the oocyte, such as *mos*, are capable of polyadenylation in a CPE-independent manner [29]. Other RNA-binding proteins besides CPEB have indeed been proposed to regulate the polyA tail length of maternal mRNAs, such as Musashi [30], Zar1l [31,32] or Hnrnpa1 [33].

While only those maternal mRNAs that contain CPEs or other motifs in optimal contexts can escape deadenylation during oogenesis, cytoplasmic polyadenylation becomes more permissive after the first embryonic cleavages (Figure 1) [20,34]. In contrast, deadenylation becomes more restrictive, and only mRNAs containing specific sequence motifs within the 3′ UTR are deadenylated [20,22,35]. Both PARN [36] and the CCR4-NOT complex, aided by the adaptor protein BTG4, are responsible for this second wave of deadenylation in the embryo [37–39].

How do CPEs promote polyadenylation? Depending on its phosphorylation state, CPEB has been proposed to recruit either PARN or the polyA polymerase GLD2 (also known as TENT2, PAPD4 or Wispy) to promote deadenylation or polyadenylation of CPE-containing mRNAs, respectively [40,41]. However, the ability of CPEB to mediate deadenylation has been challenged by recent data showing that there is no preferential deadenylation of CPE-containing mRNAs during frog oogenesis and embryogenesis [20]. Regarding polyadenylation, Xenopus Gld2 has been reported to polyadenylate maternal mRNAs *in vitro* in the presence of CPEB, CPSF and another factor, Symplekin [40]. While Gld2 is essential for Drosophila development [42], it is dispensable for mouse oogenesis [43], where the canonical polyA polymerase PAPα (also known as PAPOLA) has been implicated in polyadenylation [44]. Indeed, both canonical and Gld2 polymerases contribute to maternal mRNA polyadenylation in flies [45], a possibility that may extend to other species.

In addition to the change in length, the polyA tails of mouse and fish maternal mRNAs have been reported to contain different nucleotides [46,47]. TENT4A and TENT4B have recently been implicated in the incorporation of guanosines [46], which may stabilize the newly synthesized polyA tails [48]. In contrast, TUT4 and TUT7 incorporate uraciles and have been associated with mRNA clearance [49,50]. TUTs may also play a role in maternal mRNA activation, as uridylated mRNAs are subject to polyadenylation [46] and the depletion of TUT4 and TUT7 in mouse oocytes results in the accumulation of deadenylated mRNAs [50]. In addition, maternal mRNAs undergo partial 3′ UTR degradation, a process that promotes the activation and polyadenylation of certain mRNAs [46,51,52].

**Figure 2.**
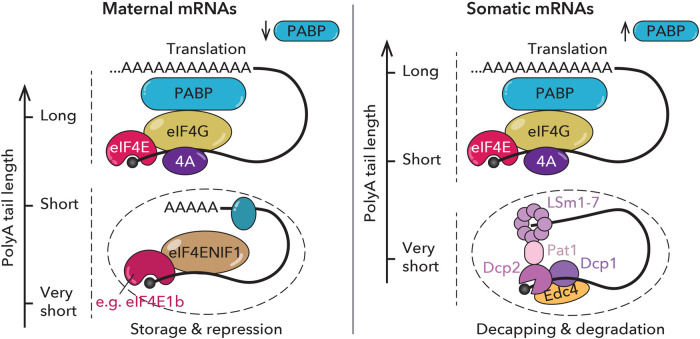
PolyA tail length of maternal mRNAs correlates with translation efficiency but not with stability. Due to limited amounts of polyA binding protein (PABP) in the oocyte, polyA tail length correlates with translation efficiency during early development. In somatic cells, where PABP is abundant, polyA tail length does not correlate with translation efficiency. While mRNAs with polyA tails shorter than 10–12 nucleotides are decapped and degraded in somatic cells, deadenylated maternal mRNAs can be stabilized in RNA granules (dashed circles) by RNA-binding proteins, such as eIF4E1b in vertebrates.

It is only during oogenesis and early embryogenesis that regulation of the polyA tail length of maternal mRNAs allows fine-tuning of translation. Why? In general, the polyA tail plays a role in cap-dependent translation, where the 5′ and 3′ ends of an mRNA are recognized by the mRNA cap-binding protein eIF4E and the cytoplasmic polyA-binding protein PABPC, respectively [53–55]. While PABPC is an abundant protein in somatic cells and allows translation of mRNAs with short polyA tails [17,56,57], a study in frogs suggests that PABPC levels are limiting in the oocyte, leading to the preferential binding of PABPC to mRNAs with long polyA tails (Figure 2) [58]. Consistent with this, overexpression of PABPC in oocytes promoted translation of mRNAs with short polyA tails, suggesting that limiting PABPC levels contribute to the coupling of polyA tail length and translation efficiency [58].

## RNA modifications

Two RNA modifications, namely 5-methylcytosine (m^5^C) and N^6^-methyladenosine (m^6^A), have been reported to accumulate at different levels in maternal mRNAs during development. While these modifications have mainly been implicated in the regulation of maternal mRNA stability, some of the proteins that bind to m^5^C or m^6^A can also affect translation.

Maternal mRNAs from several species have been reported to be decorated with m^5^C [59]. In zebrafish, the number of mRNAs containing m^5^C is high in early embryos and decreases between 4 and 6 hpf [60], a time when many maternal mRNAs are degraded [4]. m^5^C modifications may thus contribute to maternal mRNA stability, for example by recruiting Y-box proteins (YBXs) [60–62]. Both YBX1 and YBX2 (also known as MSY2 in mice) have been implicated in maternal mRNA storage [60,63–65], and YBX2 has been shown to be essential for oogenesis and spermatogenesis in mice [66]. However, YBX2 phosphorylation switches the function of YBX2 from mRNA storage to degradation [64]. YBX2 is also able to repress translation *in vitro* [67], suggesting a role for m^5^C in regulating not only maternal mRNA stability but also translation.

m^6^A modifications can recruit RNA-binding proteins that induce either mRNA storage or degradation. YTHDF proteins promote the clearance of m^6^A-containing mRNAs and are essential for zebrafish oogenesis [68–70]. In contrast, IGF2BPs have been reported to prevent the degradation of mRNAs containing GG(m^6^A)C motifs [71]. In particular, Igf2bp3 stabilizes maternal mRNAs in zebrafish [72] and associates with translationally repressed mRNAs in somatic P-bodies [73]. Furthermore, m^6^A and m^5^C have been proposed to play opposing roles in the formation of cytoplasmic condensates containing maternal mRNAs, the RNA-binding protein Rbm14 and the deadenylase PARN. While m^6^A promoted condensate formation and PARN activity, m^5^C antagonized it [74]. Investigating how m^6^A and m^5^C modifications are specifically deposited will be key to understanding their effects on translation and mRNA stability during development.

## RNA-binding proteins inhibiting translation

Initiation is the rate-limiting step of translation and relies on the assembly of the eIF4F complex at the 5′ end of an mRNA. Therefore, disruption of eIF4F assembly by RNA binding proteins is a common strategy to inhibit translation of both somatic and maternal mRNAs.

eIF4F consists of the mRNA cap-binding protein eIF4E, the scaffolding protein eIF4G, and the RNA helicase eIF4A [53] (Figure 2). While eIF4E interacts directly with eIF4G, this interaction can be inhibited by eIF4E binding proteins (eIF4EBPs), which block the binding surface for eIF4G on eIF4E [75]. eIF4EBPs are targets of the mTOR kinase, which phosphorylates eIF4EBPs under normal growth conditions to prevent their association with eIF4Es [76]. While mTOR activity is high in the mature oocyte, it decreases after fertilization [77]. Thus, in the early embryo, unphosphorylated eIF4EBPs may bind to eIF4Es and repress translation, yet other mechanisms must exist to keep translation repressed in the mature oocyte.

In the late 1990s, Xenopus CPEB was reported to interact with eIF4E through the adaptor protein Maskin (also known as TACC3) [78]. In the proposed model, Maskin interferes with the ability of eIF4E to bind to the mRNA cap and to initiate translation. However, Maskin lacks the characteristic eIF4E-binding motif found in other eIF4E-binding proteins, making this interaction questionable. Furthermore, studies in mammals have reported a role for TACC3 in the cytoskeleton rather than in translation [79,80].

Other eIF4E-binding proteins have been reported to interact with eIF4Es in the germline and to repress translation, such as Drosophila Cup [81] or *Caenorhabditis elegans* IFET-1 [82]. Vertebrates have evolved a germline-specific paralog of the cap-binding protein eIF4E, known as eIF4E1b [83–86]. Unlike canonical eIF4Es, eIF4E1b does not interact with eIF4G and therefore cannot participate in translation [86]. eIF4E1b mRNA targets contain short polyA tails and low translational efficiency in the zebrafish embryo [86]. By blocking access of decapping enzymes to the mRNA cap, eIF4E1b may store maternal mRNAs with short polyA tails that are normally degraded in somatic cells [86,87] (Figure 2). Several RNA-binding proteins that also associate with eIF4E1b-bound mRNAs, including Zar1 and Zar1l, Lsm14b [88] and Patl2 [89], have also been implicated in the repression of maternal mRNAs. However, the specific contribution of these proteins to the regulation of maternal mRNA dormancy remains to be elucidated.

## RNA granules

During oogenesis and embryogenesis, mRNAs can localize to RNA granules, membraneless compartments composed of RNAs and proteins with diverse cellular functions. For example, processing bodies (P-bodies) have been proposed as sites of translational repression as they lack ribosomes and most translation factors [90]. While somatic P-bodies contain proteins involved in mRNA decay [91], several studies suggest that P-bodies function in mRNA storage [90,92]. Of particular interest for maternal mRNA dormancy, P-bodies lacking mRNA decay proteins or containing proteins that impair mRNA decay have been reported in oocytes of several species.

In *C. elegans*, maternal mRNAs accumulate during oogenesis in specialized granules containing the P-body component CGH-1, an ortholog of human DDX6, but not the decapping component PATR-1 [93]. In flies, P-bodies located at the posterior pole of late stage oocytes (stages 9 and 10) contain only the decapping cofactor dDcp1, but not the decapping enzyme Dcp2 or the exonuclease Pacman [94]. Moreover, in flies, P-bodies play a role in the selective translation of mRNAs involved in embryonic patterning [95]. For example, the Drosophila CPEB ortholog Orb localizes to the periphery of oocyte P-bodies, where it promotes *gurken* mRNA translation, whereas mRNAs at the core of P-bodies are translationally repressed [59]. The low levels of CPEB in nurse cells, where *gurken* is transcribed, ensure that *gurken* translation occurs only in the oocyte [59].

In vertebrates, P-bodies have been shown to be absent from mouse [96] and zebrafish [86] oocytes. In frog oocytes, RNA granules termed localization-bodies (L-bodies) localize to the vegetal pole and contain P-body components (such as Lsm14b or eIF4ENIF1) as well as translation factors (such as eIF3a and eEF1a2) that are normally absent from P-bodies [97]. L-bodies may contribute to the storage of maternal mRNAs at the vegetal pole of frog oocytes, where they must later be translated to ensure embryonic patterning. Mature mouse oocytes contain subcortical RNA aggregates containing some P-body components, such as DDX6, but lacking the decapping cofactor DCP1A [96]. In zebrafish, P-bodies reassemble upon egg activation [86]. Although mRNA decapping components localize to embryonic P-bodies, eIF4E1b may stabilize mRNAs in P-bodies by binding to the mRNA cap [86] (Figure 2). The localization of eIF4E1b to P-bodies is driven by its interaction with eIF4ENIF1 (also known as 4E-T) [86], which has also been reported to stabilize mRNAs in somatic cells when bound to eIF4E [98].

In mammalian oocytes, a recent study showed that translationally repressed mRNAs with long polyA tails are stored in mitochondria-associated membraneless compartments (MARDOs) [99]. MARDO formation depends on an increase in mitochondrial membrane potential and on the RNA-binding protein ZAR1, which was previously reported to repress maternal mRNAs. Mitochondria also localize to RNA-containing membraneless compartments in early oocytes, the so-called Balbiani bodies [100]. Although both Balbiani bodies and MARDOs contain mitochondria and mRNAs, they form at different stages of oogenesis and differ in their physical properties and composition [99,101]. While Balbiani bodies may be involved in the selection of healthy mitochondria during early oogenesis [102], the clustering of mitochondria in MARDOs may be important for minimizing the production of reactive oxide species in late oocytes [99]. The conservation of MARDO beyond mammals remains elusive.

While RNA granules can influence the translation and lifespan of mRNAs, little is known about the mechanisms that select which mRNAs can enter or exit these granules. Studying these mechanisms may lead to a better understanding of how mRNAs transition from an inactive to an active state, and how they are ultimately degraded during development.

## MicroRNAs

In addition to proteins, miRNAs can also regulate maternal mRNA fate. MiRNAs are small RNAs with complementarity to mRNA sequences that can promote the translational repression or degradation of target mRNAs [103]. In zebrafish, miR-430 is produced at the onset of ZGA and induces first repression (at 4 hpf) and then degradation of target mRNAs by promoting deadenylation [104,105]. Later in development, miR-430 is responsible for clearing ∼20% of maternal mRNAs that are degraded by binding to the 3′ UTR of target mRNAs [104]. In Xenopus, miR-427, an ortholog of miR-430, also promotes deadenylation-mediated degradation of maternal mRNAs [106]. In mice, miRNAs have also been reported to be important for early embryonic development, as oocytes depleted of miRNAs cannot progress through the first cell division [107].

## mRNA-independent mechanisms of dormancy

Maternal mRNA regulation is not the only mechanism that contributes to translational repression in the oocyte. In addition to mRNAs, oocytes also store other components necessary for translation, including ribosomes. Like mRNAs, maternal ribosomes are produced during oogenesis to sustain translation during early embryonic development and must be stored in a repressed state for long periods of time. To accomplish this, maternal ribosomes associate with four conserved factors, namely eEF2, Habp4, eIF5a and Dap1b/Dapl1 (or its paralog Dap), that bind to functionally important sites of the ribosome and contribute to their storage and repression [10]. The four factors dissociate from maternal ribosomes during embryonic development by a yet unknown mechanism, correlating with an increase in translation [10]. While this dormant state of maternal ribosomes exists in Xenopus and zebrafish [10], its conservation in mammals remains unclear.

In addition to ribosomes, core translation factors are deposited in the oocyte and regulate translation during early embryonic development [108]. eIF2α is a component of the eIF2 complex that delivers the first methionyl-tRNA to the small subunit of the ribosome [53]. Phosphorylation of eIF2α inhibits the exchange of GDP for GTP in the eIF2 complex, which is necessary for the loading of Met-tRNA onto eIF2 [109]. eIF2α is phosphorylated in the mature oocyte and dephosphorylated upon fertilization [110,111], correlating with an increase in translation during embryogenesis. Phosphorylation of the translational elongation factor eEF2 also regulates translation. In particular, phosphorylation of eEF2 by eEF2 kinase (eEF2K) inhibits the translocation of peptidyl tRNAs from the A to the P site of the ribosome [112,113]. In the mouse oocyte and embryo, eEF2K-mediated phosphorylation of eEF2 oscillates in a cell cycle-dependent manner, with phosphorylation occurring outside of M phase [114]. In sea urchin embryos, eEF2 phosphorylation also oscillates with the cell cycle for one of the two eEF2 isoforms [115]. eEF2 phosphorylation may therefore contribute to translational repression in the embryo while allowing translation of specific mRNAs during mitosis.

## Discussion

**Figure 3.**
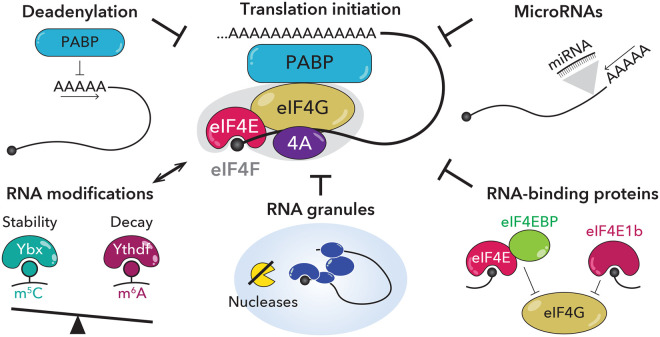
Basic mechanisms of maternal mRNA dormancy. As in many other contexts, translational repression during early development depends on inhibiting the assembly of the eIF4F complex (composed of eIF4E, eIF4G and eIF4A; center) on the mRNA cap. Repressive mechanisms can act on the mRNA itself (e.g. by deadenylation or addition of specific RNA modifications; left) or by RNA-binding proteins or microRNAs (right). In addition, some proteins can target mRNAs to specialized RNA storage granules depleted of ribosomes and factors essential for translation (bottom).

In this review, we summarize the multiple mechanisms that exist to repress and co-ordinate the translation of maternal mRNAs at a given time during development. While those we focus on mostly share the common goal of blocking the assembly of the eIF4F complex on an mRNA (Figure 3), others interfere with the assembly of the 43S preinitiation complex or ribosome function. It is important to note that the mechanisms described do not operate in isolation. For example, RNA modifications can affect RNA granule formation [74], and proteins that interfere with eIF4F assembly or binding to the mRNA cap can sense the length of the polyA tail at the 3′ end (e.g. eIF4E1b binds to mRNAs with reported short polyA tails [86]).

In addition to being susceptible to translational control, maternal mRNAs must be stable in order to control gene expression in the early embryo. Translation has been associated with a shorter mRNA lifespan for both maternal [6] and somatic [116] mRNAs, and proteins involved in translational repression can also influence mRNA stability [32,71]. Just as unused shoes remain as good as new, avoiding translation of maternal mRNAs may extend their lifespan.

While this review focuses on maternal mRNA dormancy, similar mechanisms may apply in other cellular contexts. For example, in neurons, synaptic plasticity requires rapid changes at the protein level that cannot be achieved by modulating transcription. As in embryos, CPEB mediates polyadenylation and subsequent translation of specific mRNAs at synapses [117]. Furthermore, in cancer, oncogene mRNAs have been shown to be stabilized by YBX1 binding to m^5^C modifications [61]. Therefore, the study of maternal mRNA dormancy may have broad implications in other biological contexts beyond development.

## Perspectives

- Maternal mRNAs are the only templates for gene expression during the oocyte-to-embryo transition. Proper regulation of maternal mRNA translation and stability is key to fertility and normal embryo development.
- There are many mechanisms acting at different levels to regulate translation of maternal mRNAs, and new ones are likely to be discovered. In addition, little is known about the selectivity of these mechanisms and how they work together.
- Multidisciplinary approaches will be needed to understand how factors described years ago act at the mechanistic level. The oocyte-to-embryo transition is an ideal system to study post-transcriptional mechanisms of gene expression regulation, and thus how mRNAs and translation can be regulated in different contexts.

## Abbreviations

CPE: cytoplasmic polyadenylation element
CPSF: Cleavage and Polyadenylation Specificity Factor
PAS: polyadenylation signal
UTR: untranslated region
ZGA: zygotic genome activation
miRNAs: microRNAs
